# Study of changes in lipid profile and fasting blood glucose in protease inhibitor exposed HIV/AIDS patients in School of Tropical Medicine, Kolkata

**DOI:** 10.1186/1471-2334-12-S1-P34

**Published:** 2012-05-04

**Authors:** Ananya Bhowmik, Rajyasree De, Manotosh Mahato, Sujit Kumar Das, Subhasish Kamal Guha

**Affiliations:** 1 Centre of Excellence in HIV, School of Tropical Medicine, Kolkata, India; 2 Department of Tropical Medicine, School of Tropical Medicine, Kolkata, India

## Background

The national second-line Anti retroviral Therapy (ART) programme was started in Kolkata in December 2008. It included a combination of Tenofovir, Lamivudine and Ritonavir-boosted Lopinavir ± Zidovudine. Dyslipidaemia and increased fasting blood sugar (FBS) often complicate protease inhibitor-containing ART. Thus a prospective study was designed to observe the above changes.

## Methods

The data of 48 patients, on protease inhibitor for one year were analyzed. Body Mass Index (BMI), grip strength (GS), Triceps skin fold (TSF), 24 hour dietary recall, serum triglyceride (TG), total cholesterol (TC), HDL, LDL, VLDL and FBS were estimated for all patients at baseline, 6 months and after one year.

## Results

There was a significant increase in TG, TC and VLDL levels at 1 year as compared to baseline (p=0.013, 0.00 and 0.00 respectively) whereas LDL significantly increased at 6 months only (p=0.029). HDL decreased significantly at 6 months (p=0.019). TSF significantly decreased both at 6 and 12 months (p=0.00 and 0.00 respectively). The BMI and GS showed a significant increase at both 6 months (p=0.001, 0.000 respectively) and 1 year (p=0.005 and 0.00 respectively). Four patients with normal baseline FBG and one with impaired fasting glucose progressed to overt diabetes (FBG > 124 mg/dl) at 12 months. No significant change was noted in energy and protein intake of patients.

## Conclusion

There is an increased incidence of dyslipidaemia and unmasking of diabetes related to protease inhibitor in this cohort. There has been an improvement in nutritional status as shown by BMI and GS.

